# Between Tics and Obsessions: Case Report and Review on OCD-Tic comorbidity

**DOI:** 10.1192/j.eurpsy.2026.10439

**Published:** 2026-07-31

**Authors:** P. M. Oliveira, B. Moura

**Affiliations:** Psychiatry Department, Gaia and Espinho Local Health Unit, Vila Nova de Gaia, Portugal

## Abstract

**Introduction:**

Tic disorder and Obsessive Compulsive Disorder (OCD) are neuropsychiatric conditions that can occur simultaneously and share a waxing and waning course. Both disorders involve dysfunction in cortico-striatal-thalamo-cortical (CSTC) circuits and share overlapping phenomenology, including repetitive, intrusive, and irresistible behaviors.

**Objectives:**

To illustrate the clinical complexity of the OCD-Tic comorbidity by presenting a clinical case of a young male meeting both diseases’ diagnostic criteria.

**Methods:**

Single-case report and narrative review of relevant literature of the comorbidity of OCD-Tic disorder.

**Results:**

A 20-year old male presented with simple motor tics and sexually themed-related obsessions and mental compulsions causing marked impairment in social and academic functioning since he was 8 years old. Pharmacological management was started with sertraline, titrated up to 150 mg/day, combined with risperidone up to 2 mg/day, leading to significant clinical improvement. The treatment was well tolerated, and both obsessive-compulsive symptoms and tics were notably and persistently reduced.

OCD comorbid with tic disorder enter a specific category in DSM classification since they have a worse prognosis and tend to be more frequent in male-individuals with OCD onset in childhood. Genetics and neuroinflammation may explain the occurrence of these disorders.

**Conclusions:**

This case highlights the clinical relevance of recognizing and addressing comorbidity between tic disorders and OCD. Shared neurobiological substrates, particularly dysregulation in dopaminergic and serotonergic pathways within CSTC circuits, may explain the overlap. Combined pharmacological treatment with SSRI and atypical antipsychotics is clinically beneficial. Precocious identification and an integrated treatment approach are essential to address properly the OCD–Tic comorbidity.

**Disclosure of Interest:**

None Declared

